# Increased expression of proenkephalin and prodynorphin mRNAs in the nucleus accumbens of compulsive methamphetamine taking rats

**DOI:** 10.1038/srep37002

**Published:** 2016-11-14

**Authors:** Jean Lud Cadet, Irina N. Krasnova, Donna Walther, Christie Brannock, Bruce Ladenheim, Michael T. McCoy, Daniel Collector, Oscar V. Torres, Ndeah Terry, Subramaniam Jayanthi

**Affiliations:** 1 Molecular Neuropsychiatry Research Branch, DHHS/NIH/NIDA Intramural Research Program, 251 Bayview Boulevard, Baltimore, MD 21224, USA

## Abstract

Addiction is associated with neuroadaptive changes in the brain. In the present paper, we used a model of methamphetamine self-administration during which we used footshocks to divide rats into animals that continue to press a lever to get methamphetamine (shock-resistant) and those that significantly reduce pressing the lever (shock-sensitive) despite the shocks. We trained male Sprague-Dawley rats to self-administer methamphetamine (0.1 mg/kg/infusion) for 9 hours daily for 20 days. Control group self-administered saline. Subsequently, methamphetamine self-administration rats were punished by mild electric footshocks for 10 days with gradual increases in shock intensity. Two hours after stopping behavioral experiments, we euthanized rats and isolated nucleus accumbens (NAc) samples. Affymetrix Array experiments revealed 24 differentially expressed genes between the shock-resistant and shock-sensitive rats, with 15 up- and 9 downregulated transcripts. Ingenuity pathway analysis showed that these transcripts belong to classes of genes involved in nervous system function, behavior, and disorders of the basal ganglia. These genes included prodynorphin (PDYN) and proenkephalin (PENK), among others. Because PDYN and PENK are expressed in dopamine D1- and D2-containing NAc neurons, respectively, these findings suggest that mechanisms, which impact both cell types may play a role in the regulation of compulsive methamphetamine taking by rats.

Methamphetamine is a highly addictive psychostimulant whose abuse is characterized by repeated use of the drug despite adverse medicolegal ramifications^1^. The inability to quit taking methamphetamine is probably related to persistent neuroadaptive changes in brain regions thought to be responsible for rewarding effects of drugs and cognitive processes that regulate habitual behaviors^2^,^3^. These neuroadaptations are thought to include altered synaptic plasticity, transcriptional alterations, and epigenetic changes in mesostriatal and mesocorticolimbic projection areas such as the nucleus accumbens (NAc)^4^,^5^,^6^. Despite the cognitive and medical ravages associated with addiction to the drug, much remains to be done in order to develop adequate treatment to these patients.

In order to develop more rational approaches to treat methamphetamine addiction, it is important to delineate the neurobiological substrates of acquisition and persistent abuse of the drug. Rat self-administration models are often used to study potential molecular bases of drug taking behaviors by humans^7^. Methamphetamine self-administering rats, given extended access to the drug, escalate their use over time^8^. This approach has helped to discover the potential role of various neurotransmitter systems, the involvement of gene regulation, and epigenetic mechanisms in the modulation of methamphetamine-taking behaviors^9^,^10^,^11^,^12^. Importantly, however, these studies have used all animals that self-administered methamphetamine, whereas many human substance users reduce or stop their drug use in the presence of potential adverse consequences and do not develop addiction^13^,^14^. In contrast, individuals who become addicts increase the dose and frequency of drug use and continue to do so in spite of negative life events associated with their continuous drug abuse.

The present study was thus carried out to identify transcriptional changes in the NAc of rats that showed two divergent phenotypes while receiving contingent footshocks during methamphetamine self-administration^15^. One group of animals continued to compulsively press a lever to receive the drug (shock-resistant) whereas another reduced their drug intake (shock-sensitive). Here we report that the shock-resistant rats showed significant increases in prodynorphin (PDYN) and proenkephalin (PENK) mRNA levels in the NAc. These results further implicate these neuropeptides in the manifestation of methamphetamine addiction.

## Results

### Behaviors

**Figure 1:**
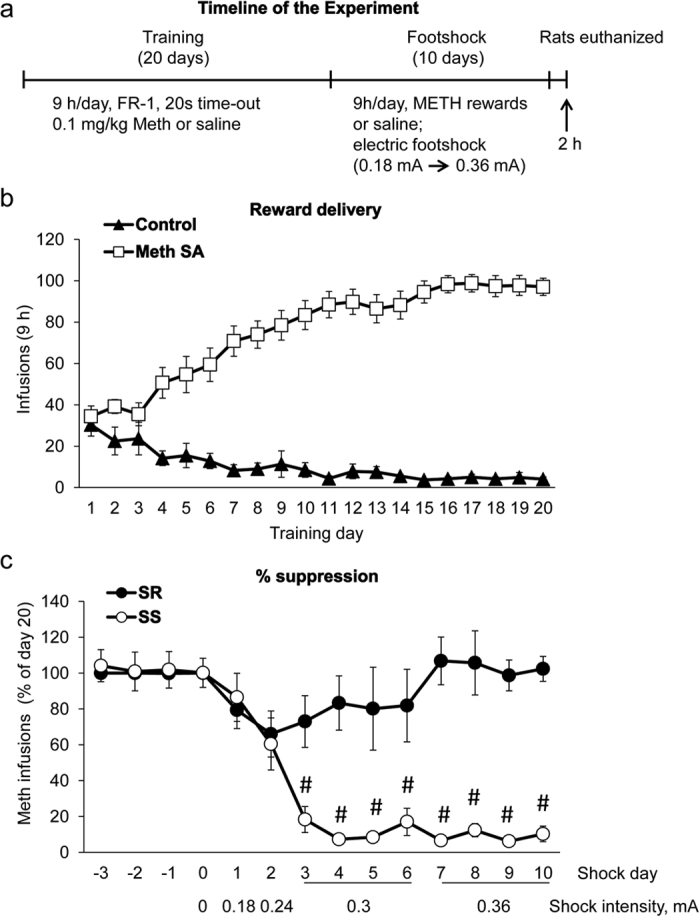
Effects of prolonged methamphetamine self-administration and contingent footshocks in rats. (**a**) Timeline of the experiment. (**b**) Escalation of methamphetamine intake during the training phase of the experiment. N = 6 (control group) and 12 (methamphetamine self-administration group). (**c**) Suppression of lever pressing by footshocks in shock-sensitive (SS) rats but not in shock-resistant (SR) compulsive methamphetamine takers. N = 6 per group. Data are means + SEM of infusions (**b**) or percentage suppression (**c**). During the footshock phase, shock intensity was increased from 0.18 to 0.36 mA, with 50% of rewarded lever presses being accompanied by footshocks. ^#^Significantly different from the SR group, p < 0.001.

The timeline for the behavioral experiments is shown in Fig. 1a. The methamphetamine-trained rats (n = 12) increased their drug intake and control rats (n = 6) decreased their saline intake per day during self-administration training (Fig. 1b). The repeated measures ANOVA for infusions earned included the between-subject factor reward type (saline, methamphetamine) and the within-subject factor of self-administration day (training days 1–20). Analysis showed a significant effect of training day x reward type [F_(19,304)_ = 20.2, p < 0.001]. The significant interaction reflects that for methamphetamine-trained rats drug intake continued to increase for the first 12 self-administration days, while intake decreased and stabilized after seven days for the saline rats.

During the punishment phase, we increased shock intensity from 0.18 to 0.36 mA over a period of 10 days (Fig. 1c). As the level of footshocks increased, reinforced responding decreased in shock-sensitive, but not in shock-resistant rats (Fig. 1c). We classified 6 out of 12 methamphetamine self-administering rats as shock-resistant because they showed less than 20% decrease in the number of methamphetamine infusions from pre-shock level (Fig. 1c). In contrast, we classified 6 animals as shock-sensitive because they showed more than 80% suppression of drug infusions on shock days 7–10 in comparison to baseline (Fig. 1c). The statistical analysis of methamphetamine infusions earned included the between-subject factor of group (shock-resistant, shock-sensitive) and within-subject factor of shock day (shock days 1–10). Analysis showed a significant effect of shock day x group [F_(9,90)_ = 8.6, p < 0.001)], indicating that shock-resistant rats self-administered more methamphetamine infusions than shock-sensitive animals, an effect that was most pronounced at 0.36 mA shock level (Fig. 1c). We found that, during the punishment phase, shock-resistant rats received 487 ± 44 shocks, while shock-sensitive rats received 116 ± 17 shocks [F_(1,10)_ = 61.78, p < 0.001].

### Affymetrix arrays

**Figure 2:**
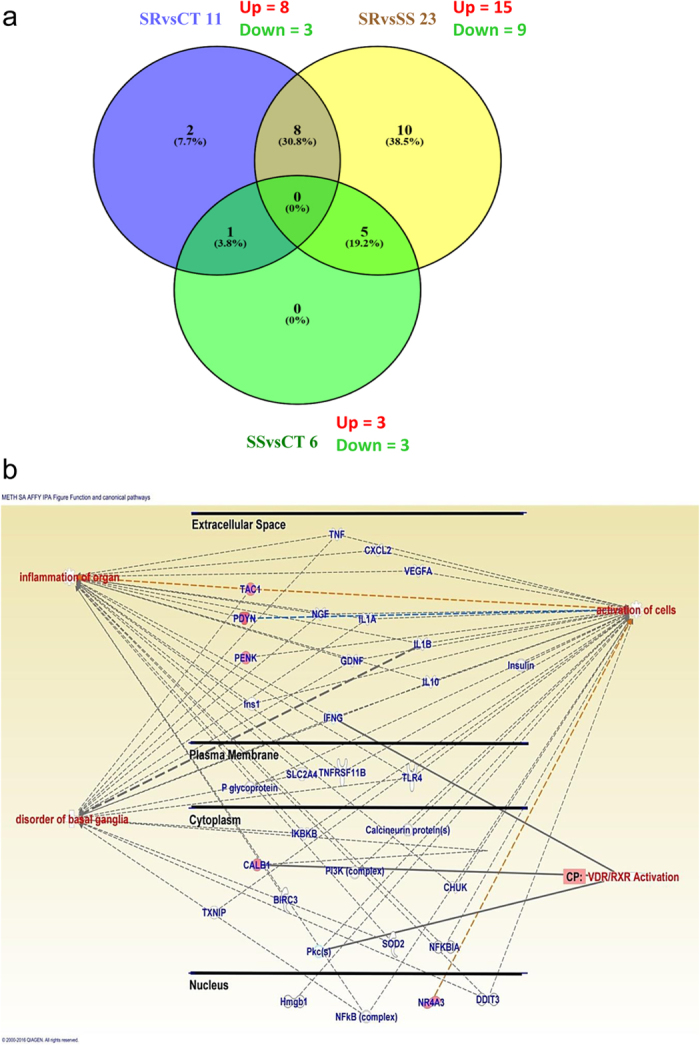
Microarray analysis of gene expression measured in the rat NAc at 2 hours after cessation of methamphetamine self-administration and footshocks. (**a**) Comparison of microarray results of pairwise comparisons between shock-resistant and control (SRvsCT), shock-resistant and shock-sensitive (SRvsSS), and shock-sensitive and control (SSvsCT). There were 11, 23, and 6 differentially expressed genes in the SRvsCT, SRvsSS, and SSvsCT comparisons, respectively. The number of up- and down-regulated genes for each comparison is shown in red and green, respectively. (**b**) Ingenuity pathway analysis shows that differentially expressed genes in the SRSS comparison are involved in activation of cells and disorders of the basal ganglia. The networks and functional analyses were generated through the use of QIAGEN’s Ingenuity Pathway Analysis (IPA®, QIAGEN Redwood City, www.qiagen.com/ingenuity).

In order to investigate transcriptional changes associated with compulsive methamphetamine taking, we used an unbiased approach that involved the use of the Affymetrix array platform containing 68,842 probes. Of these probes, 24,753 were protein coding, 28,724 were noncoding and the rest consisted of unassigned pseudogenes, among others. The array data have been deposited into the Gene Expression Omnibus (GEO) database of the National Center for Biotechnological Information, NIH, with NCBI tracking system #18028247. Figure 2 and Table 1 show the results of these array experiments. There were three pairwise comparisons: shock-resistant versus control (SRvsCT), shock-sensitive versus control (SSvsCT), and shock-resistant versus shock-sensitive (SRvsSS) groups. We found that 40 transcripts were differentially affected (±1.7 fold change, p < 0.05) in the NAc 2 h after the last self-administration session. As shown in Fig. 2a, there were 11 genes (8 up- and 3 down-regulated) with differential expression in the SRvsCT comparison, 6 (3 up- and 3 down-regulated) in the SSvsCT comparison, and 23 (14 up- and 9 down-regulated) in the SRvsSS comparison. We performed functional annotation and classification analyses for significantly changed genes using DAVID Annotation tool^16^ and supported the classification scheme by literature searches. To focus on changes that could be related to gene function, we included only genes classified using DAVID Annotation tool in further analysis and excluded all undefined genes. The names of these genes and their classification are shown in Table 1. The list includes PDYN, PENK, prostate transmembrane protein, androgen induced 1 (pmepa1), calbindin 1 (Calb1), dachsprung family transcription factor 1 (Dach1), and nuclear receptor subfamily 4, group A, member 3 (Nr4a3). Differentially expressed genes belong to several functional classes including those that participate in metabolism, signal transduction, synaptic plasticity, and transcription regulation (Table 1). Ingenuity pathway analysis shows that these genes are also involved in inflammation and disorder of the basal ganglia (Fig. 2b).

### Quantitative RT-PCR

**Figure 3:**
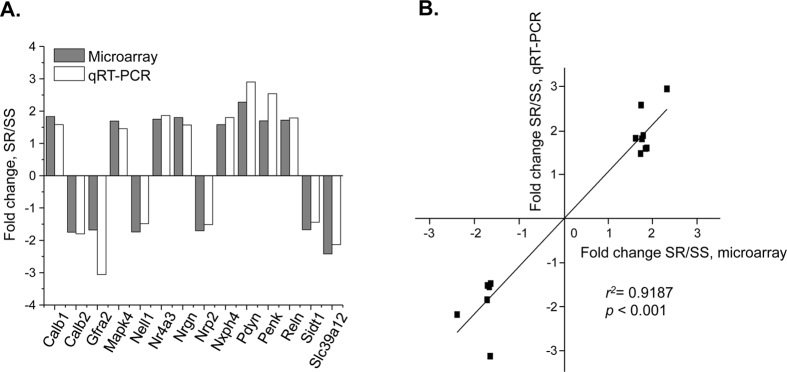
qRT-PCR validation of genes identified in the SRvsSS comparison obtained in the microarray analysis. Validation of differences in the expression of genes between shock-resistant and shock-sensitive groups obtained in microarray experiments using qRT-PCR. (**A**) The increases and decreases in the expression of 14 genes identified using microarray analysis were validated with qRT-PCR. All fold-changes found with qRT-PCR were significantly different (p < 0.05). (**B**) Differences in gene expression between shock-resistant and shock-sensitive groups obtained using microarray analysis significantly correlated with qRT-PCR data. N = 6 per group.

We then used qRT-PCR to validate the expression of 14 out of 23 genes identified in the microarray experiment as significantly changed in the SRvsSS comparison. We confirmed changes in Calb1, mitogen-activated protein kinase 4 (Mapk4), Nr4a3, neurogranin (Nrgn), neurexophilin 4 (Nxph4), PDYN, PENK and reelin (Reln) mRNA levels that were up-regulated at 2 hours after drug abstinence (Fig. 3A). We also confirmed down-regulation in the expression of calbindin 2 (Calb2), GDNF family receptor alpha 2 (Gfra2), neural EGFL-like 1 (Nell1), neuropilin 2 (Nrp2), SID1 transmembrane family, member 1 (Sidt1) and solute carrier family 39, member 12 (Slc39a12) in the NAc of shock resistant versus shock-sensitive rats (Fig. 3A). In order to quantify potential relationships between the microarray and qRT-PCR data (Fig. 3A), we performed a regression analysis and found a strong positive correlation [F_(1,12)_ = 147.8, p < 0.001] between changes in gene expression obtained by both methods (Fig. 3B). This analysis validates the inclusion criteria for choosing genes with altered expression using the discovery Affymetrix array platform.

**Figure 4:**
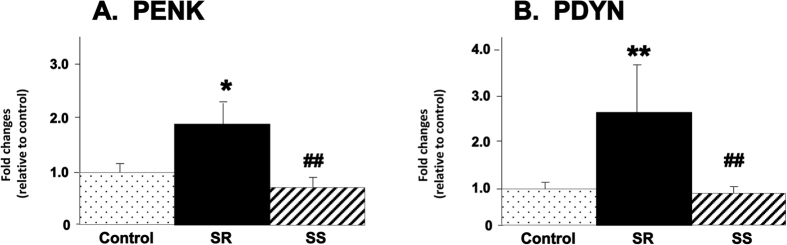
Effects of methamphetamine self-administration and footshocks on the expression of PDYN and RENK expression in the NAc. qPCR experiments were conducted using individual RNA samples from the NAc of rats that fell within the 3 groups. Values are means + SEM in comparison to the control animals. The shock-resistant phenotype showed significant increases in (**A**) PDYN and (**B**) PENK mRNA levels, replicating the microarray data. N = 6 per group. *p < 0.05, **p < 0.01, in comparison to the control group; ^##^p < 0.01, in comparison to the shock-resistant phenotype.

Figure 4A shows the results of qRT-PCR for PDYN and PENK gene expression. There were significant increases in PDYN in the shock-resistant group [F_(2, 15)_ = 10.29, p = 0.0015] (Fig. 4A). PDYN mRNA expression was higher in the shock-resistant group than in the control (+2.7-fold) and shock-sensitive (+2.9-fold) groups. We also found that PENK expression was increased in the shock-resistant group [F_(2.15)_ = 6.87, p = 0.0076], showing significant increases in comparison to control (+1.89-fold) and shock-sensitive (+2.54-fold) groups (Fig. 4B). Because PDYN and PENK are expressed in respective dopamine receptor D1(DRD1)- and dopamine receptor D2 (DRD2)-containing neurons^17^,^18^, we sought to determine if compulsive methamphetamine self-administration was also accompanied by changes in the expression of the five subtypes of dopamine receptors. We found no significant changes in dopamine receptors in the NAc (Supplementary Figure 1S), therefore implicating opioid-dependent signals within intrinsic neurons in the long-term manifestations of compulsive methamphetamine intake.

**Figure 5:**
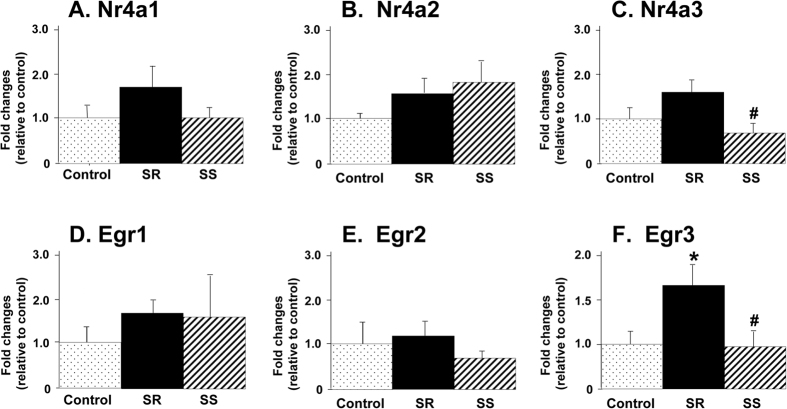
Effects of methamphetamine self-administration and footshocks on the expression of Nr4a and Egr classes of immediate-early genes. qRT-PCR experiments were conducted as described in Fig. 4. Values are means + SEM in comparison to the control animals. (**A,B**) There were no significant changes in the expression of Nr4a1 and Nr4a2 between the groups. (**C**) The shock-resistant rats showed significantly higher Nr4a3 expression than the shock-sensitive animals. (**D,E**) There were no significant differences in the expression of Egr1 and Egr2 expression between the 3 groups. (**F**) The shock-resistant phenotype showed higher Egr3 expression than the control and shock-sensitive phenotype. N = 6 per group. *p < 0.05, in comparison to the control group; ^#^p < 0.05, in comparison to the shock-resistant SR rats. Note the differences in the y-axis scale for Egr3 expression in comparison to the other genes in the figure.

The microarray analysis also identified Nr4a3 as showing significant differences (+1.7-fold) between shock-resistant and shock-sensitive groups. We used qRT-PCR to validate these changes and also measured the expression of other members in that class of immediate-early gens that included Nr4a1 and Nr4a2 (Fig. 5). We found no significant changes in the expression of Nr4a1 (Fig. 5A) and Nr4a2 (Fig. 5B) between the groups. However, there were significant differences [F_(2,15)_ = 4.295, p = 0.0335] between shock-resistant and shock-sensitive groups in Nr4a3 mRNA levels (+1.86-fold) (Fig. 5C), thus replicating the array findings.

Because a previous study had reported that acute methamphetamine administration increased the expression of early growth factor 1 (Egr1) in the rat striatum in conjunction with PDYN and PENK mRNA levels^19^, we wondered if this class of immediate-early genes that include Egr1-Egr3^20^ might also be affected in the NAc of shock-resistant rats. To test this idea, we measured Egr1-Egr3 mRNA levels (Fig. 5). We found no significant changes in Egr1 and Egr2 mRNA levels in shock-resistant and shock-sensitive groups (Fig. 5D,E). However, there were significant increases [F_(2,15)_ = 4.028, p = 0.0398] in Egr3 expression in the shock-resistant phenotype in comparison to control (+1.645) and shock-sensitive (+1.68-fold) (Fig. 5F) groups.

## Discussion

The goal of the present study was to investigate genome-wide transcriptional effects in the NAc of rats that had undergone methamphetamine self-administration and punishment that generated shock-resistant and shock-sensitive phenotypes using an unbiased approach. We showed that compulsive methamphetamine self-administration triggered increases in the expression of PDYN and PENK mRNA levels in the NAc of rats euthanized 2 hours after the last self-administration session. We used this time interval because molecular events that occur after repeated drug exposure might potentially offer a window to molecular substrates of methamphetamine addiction and its adverse sequelae. Indeed, the increased PDYN expression is consistent with the data of two recent papers that reported increases in PDYN mRNA^9^ and PDYN immunoreactivity^20^ in rats that had self-administered methamphetamine. The present observations expand on those previous reports by indicating that persistent increases in PDYN expression might be maintained only in subjects that fit an addicted phenotype but not in those that become abstinent in the presence of adverse consequences. Furthermore, our novel observations of increased PENK expression in compulsive methamphetamine takers suggest that neuropeptides localized in both DRD1- and DRD2-containing neurons^17^,^18^ might be affected in animals that continue to press a lever to obtain the drug even in the presence of footshocks. Interestingly, our observations of increased PENK are consistent with those of a previous study that reported increased NAc PENK expression in early withdrawal from cocaine self-administration^21^. Our findings are also consistent with data obtained in previous studies showing that investigator-administered methamphetamine caused an increase in PDYN expression in the NAc^16^,^22^. Importantly, an acute non-contingent methamphetamine injection increased PENK expression in the dorsal striatum but not in the NAc^19^,^22^, suggesting that PENK and PDYN may be regulated by different molecular processes when the drug is given non-contingently by investigators. In contrast, these diverse mechanisms may be activated in compulsive drug takers since we observed increased expression of both neuropeptides in the shock-resistant group. Previous findings showing that NMDA and AMPA glutamate receptor antagonists can block the effects of methamphetamine on striatal PDYN but not on striatal PENK mRNA levels^22^ also support our conclusion.

Several studies have implicated dynorphin in the process of drug addiction^23^,^24^ through its interactions with kappa opioid receptors^25^,^26^. For example, kappa opioid agonists were reported to be aversive in rodents^27^ and to decrease cocaine self-administration^28^. Interestingly, kappa opioid receptor knockout mice showed increased behavioral and biochemical responses to cocaine^29^, suggesting that endogenous kappa ligands might work to inhibit psychostimulant-induced behaviors. A role for dynorphin in methamphetamine self-administration was also shown in a paper that reported that infusion of the kappa opioid receptor antagonist, norbinaltorphine, into the NAc was able to attenuate escalation of methamphetamine intake by rats^20^. Together with our present findings, the accumulated evidence indicates that increased dynorphin expression in the case of cocaine and methamphetamine self-administration may serve to differentially regulate the biochemical and molecular events that accompany addiction to diverse psychostimulants^23^,^24^,^30^. This conclusion is consistent with the fact that there were no significant changes in the expression of PDYN in the shock-sensitive rats.

Enkephalin has also been implicated in the effects of psychostimulants including methamphetamine^19^,^22^,^31^, probably via interactions with dopamine systems since blockade of dopamine receptors by haloperidol^32^,^33^,^34^ and dopamine depletion by reserpine^35^ or 6-hydroxydopamine^36^,^37^ increased PENK mRNA levels in the rat striatum and NAc. Interestingly, prefrontal corticostriatal glutamatergic projections appear to play a significant role in maintaining increased PENK mRNA levels the rat striatum^38^. These findings are consistent with the notion that dopamine may exert an inhibitory control, whereas glutamate appears to be a stimulatory regulator of enkephalin biosynthesis^19^,^22^,^32^,^39^. Thus, our results suggest a potential decrease in dopaminergic tone and/or an increase in glutamate tone in the shock-resistant rats that showed increased PENK expression in the NAc. These ideas are consistent with the demonstration that rats that had undergone a 2 h/day methamphetamine self-administration procedure exhibited increased baseline glutamate levels in the NAc after one day of withdrawal^40^. Since we used a longer access (9 h/day) to methamphetamine self-administration in our experiments^15^,^41^, it is conceivable that rats in the present study might show even greater changes in baseline glutamate levels in the NAc. Although these ideas will need to be tested further, they are consistent with the accumulated evidence that glutamate systems do play important roles in addiction^42^.

It is of interest to relate this discussion to our findings of elevated Nr4a3 and Egr3 mRNA levels in the shock-resistant rats that also showed increased PENK and PDYN mRNA expression. Increased Egr3 expression in DRD1-containing neurons was reported in the NAc of rats that received cocaine non-contingently^43^ or contingently^44^. The changes in Egr3 expression may be related to learning and memory processes that are involved in addiction because Egr3 is essential to these cognitive phenomena^45^. When taken together, the similar observations in Egr3 expression after cocaine and methamphetamine administration suggest that this immediate early gene might participate in the regulation of PDYN via, potentially, increased Egr3 binding to the PDYN promoter as reported for other genes such as Ca^2+^/calmodulin-dependent protein kinase II alpha (Camk2 alpha) and cAMP response element-binding protein (CREB) after chronic cocaine administration^43^. A similar argument could be made for methamphetamine-induced increase in PENK expression in DRD2-containing neurons. The differential expression of Nr4a3 in the NAc between shock-resistant and shock-sensitive rats is also of interest because members of the orphan nuclear receptor family of transcription factors play important roles in several brain functions including neurodevelopmental processes, responses to addictive drugs, and neurodegeneration^46^,^47^,^48^. Our observation that shock-resistant rats showed higher Nr4a3 expression than shock-sensitive animals is consistent with findings of a previous paper that reported that chronic cocaine treatment caused increase in Nr4a3/Nor-1 expression only in addiction-prone Lewis rats but not in Fischer rats that showed less addiction-like behaviors^49^. Taken together, these observations suggest that animals prone to manifest compulsive drug taking may also show differential molecular neuroadaptations in the classes of transcription factors that regulate the expression of neuropeptides in the NAc.

Nevertheless, we cannot completely rule out the possibility that increases in PDYN and PENK mRNA levels in the NAc of shock-resistant versus shock-sensitive rats, may also depend, in part, on the different number of footshocks received by these animals instead of being solely a reflection of resistance to punishment. Indeed, in our study, shock-resistant rats received significantly more footshocks than the shock-sensitive animals during the punishment phase. Stress caused by electric footshocks could have led to the increases in PDYN and PENK mRNA levels observed in the shock-resistant rats. This suggestion is not supported by a previous study that reported that mild electric footshocks (0.2 mA) actually produced decreases in enkephalin levels in the rat ventral tegmental area (VTA)^50^ whereas we observed increased PENK expression in the NAc. Moreover, in contrast to the findings of that previous report^50^, another group of investigators failed to find any changes in enkephalin levels in the VTA after footshocks^51^. It has also been reported that stress resulting from repeated forced swim sessions is accompanied by release of dynorphin and activation of kappa-opioid receptors in the mouse NAc^52^,^53^. However, the short (0.5-s) mild-to-moderate (0.18–0.36 mA) electric footshocks used in our study are much less stressful than five repeated 15- and 6-min forced swim sessions used to cause stress in the studies by Lang *et al.*^52^ and Schindler *et al.*^53^. Supporting the present proposition that the changes in PDYN and PENK in the NAc are related to the addicted phenotype is a recent study that investigated the acute effects of yoked shock on neural activity in several brain regions while using a model of optogenetic self-stimulation with punishment in mice^54^. The authors observed increased cFos immunoreactivity in the prelimbic medial prefrontal cortex of the yoked shock group but detected no changes in the NAc, orbitofrontal cortex, or cingulate cortex^54^. Nevertheless, additional studies are required to elucidate the impact of stress-induced neuronal plasticity in the NAc and its role in resistance to footshocks in the rat model of methamphetamine self-administration with punishment.

It is of interest to note that our studies have found that a larger proportion of methamphetamine self-administration rats continued to self-administer methamphetamine in spite of electric foot shocks (shock-resistant rats) than those reported in cocaine self-administration papers^54^,^55^. However, our methamphetamine data are consistent with results of studies that we have run so far, in which we found that 40–50% of the rats are resistant to footshocks^15^. The differences may be related to the fact that we used methamphetamine while the others used cocaine^54^,^55^. The different behavior and punishment procedures used in the various studies might also influence the outcomes. There is indeed evidence that the manner of introduction of punishment can impact the punished responses, with sudden introduction of moderate to severe punishment producing larger suppression than progressive increases in footshocks^56^. Future studies using identical behavioral paradigm in experiments run concurrently for methamphetamine and cocaine self-administration using animals obtained from the same vendor will allow us to provide a more definite answer to the differences in the percentages of resistant and sensitive rats between cocaine and methamphetamine animals.

In summary, compulsive methamphetamine self-administering rats show increased expression of PDYN and PENK in the NAc in comparison to control and abstinent rats. These observations further implicate a role of opioid neuropeptides in the manifestation of methamphetamine addiction. Further delineation of the downstream molecular events that lead to development of the compulsive drug taking phenotype in response to these neuropeptides should help in the planning of more rational therapeutic approaches to induce and maintain abstinence in methamphetamine-addicted patients.

## Methods

### Animals and drug treatment

We used male Sprague-Dawley rats (Charles River Labs, Raleigh, NC, USA), weighing 350–400 g in the beginning of the study in all experiments. We housed animals in a humidity and temperature-controlled (22.2 ± 0.2 °C) room with free access to food and water. Our procedures followed the *Guide for the Care and Use of Laboratory Animals* (ISBN 0–309–05377–3) and were approved by the National Institute of Drug Abuse Animal Care and Use Committee.

### Intravenous surgery

We anesthetized rats with ketamine and xylazine (50 and 5 mg/kg, i.p., respectively) and inserted silastic catheters into the jugular veins, as described previously^15^,^41^. We attached the catheters to a modified 22-gauge cannula that was mounted to their skulls with dental cement. We injected buprenorphine (0.1 mg/kg, s.c.) one time after surgery to relieve pain and allowed the rats to recover for 5–7 days before methamphetamine self-administration training. During the recovery, training and punishment phases of the experiment, we flushed the catheters every 24–48 h with gentamicin (Butler Schein; 5 mg/ml) and sterile saline.

### Training and punishment phases

We performed the training procedure for methamphetamine self-administration according to previously described protocol^15^,^41^. On the first day of training, we brought rats to the self-administration room and chronically housed them in self-administration chambers. Animals had free access to food and water that were available in water bottles and feeders hanging on the walls of all self-administration chambers. We trained rats to self-administer dl-methamphetamine HCl (NIDA) during three 3-h sessions/day (the sessions were separated by 30 min off intervals) for 20 days under a fixed-ratio-1 with 20-s timeout reinforcement schedule. Presses on the retractable active lever activated the infusion pump. Active lever presses were also accompanied by a 5-s compound tone-light cue. Presses on inactive (stationary) lever had not reinforced consequences. We connected the catheters of rats to a modified cannula (Plastics One, Minneapolis, MN) attached to a liquid swivel (Instech Laboratories, Inc., Plymouth Meeting, PA, USA) via polyethylene-50 tubing that was protected by a metal spring. We trained the rats in four cycles of 5 days of methamphetamine self-administration and 2 days off in order to minimize weight loss. Body weight loss is a known side effect of methamphetamine use in laboratory animals^57^. During 2 days off rats remained housed in self-administration chambers, but we disconnected them from intravenous self-administration lines and covered the connecting cannulas with dust caps. Rats self-administered methamphetamine at a dose of 0.1 mg/kg/infusion over 3.5 s (0.1 ml/infusion)^15^,^41^. To prevent overdose, we limited the number of infusions per 3-h session to 35. Control rats self-administered saline under the same conditions. We started the self-administration sessions at the onset of the dark cycle and sessions began with the insertion of the active lever and the illumination of a red house light that remained on for the duration of the session. At the end of each 3-h session, the house light was turned off, and the active lever was retracted.

During the punishment phase, rats continued methamphetamine self-administration every day (three 3-h sessions/day separated by 30 min off intervals) under the fixed ratio-1 with 20-s timeout reinforcement schedule that was used during training. For methamphetamine-trained rats, 50% of the reinforced lever-presses also resulted in the concurrent delivery of a 0.5-s footshock through the grid floor^15^,^41^. We set the initial footshock at 0.18 mA and increased the shock intensity by 0.06 mA to a final value of 0.36 mA (a total of 10 punishment days).

### RNA preparation

Two hours after cessation of methamphetamine self-administration and footshocks, we euthanized the rats by decapitation with guillotine and isolated NAc samples from the brains. We extracted total RNA from individual NAc samples using Qiagen RNeasy Mini kit (Qiagen, Valencia, CA, USA). We assessed RNA integrity using an Agilent 2100 Bioanalyzer (Agilent, Palo Alto, CA, USA); RNA samples showed no degradation.

### Transcriptomic profiling using Affymetrix microarrays

We amplified the RNA using Affymetrix labeling according to standard Affymetrix protocols (Affymetrix, Santa Clara, CA, USA). We performed hybridization, labeling, scanning, and data extraction according to standard Affymetrix protocols. We analysed microarray data using the Signal Space Transformation–Robust Multi-Chip Analysis (SST-RMA) algorithm (Affymetrix). This algorithm applies a GC correction to the microarray data and transforms the microarray data signal to a similar signal space of other methods such as RT-PCR and RNA-Seq allowing comparison of microarray data to RT-PCR. We normalized the signal intensity distributions across all probe features on all the arrays. Then we pooled the signal intensities for all probe sets that define a gene for a given sample together into a single value. We used the overall signal value to compare gene expression changes between conditions. We used ANOVA to determine the differences in signals between conditions. We identified differentially expressed genes according to a 1.7-fold changes (p < 0.05) in pairwise comparisons using Affymetrix software. We performed functional annotation and classification of significantly changed genes using DAVID annotation tool (http://david.abcc.ncifcrf.gov)^16^.

### Quantitative RT-PCR analysis of mRNA levels

We validated microarray results for 14 out of 23 genes significantly changed in SRvsSS comparison by qRT-PCR as previously described^15^. Briefly, we reversed-transcribed individual total RNA from 6 rats per group into cDNA using Advantage RT-for-PCR kit (Clontech, Mountain View, CA, USA). We generated gene-specific PCR primers using LightCycler probe design software v. 2.0 (Roche Biosystems, Indianapolis, IN, USA) and purchased primers from the Synthesis and Sequencing Facility of Johns Hopkins University (Baltimore, MD, USA). We used High Capacity cDNA Reverse Transcription Kit (Invitrogen, Waltham, MA, USA) with a ViiA 7 instrument (Life Technologies, Waltham, MA, USA) to perform PCR. We normalized the relative amounts of mRNA in each sample to means of clathrin, ornithine decarboxylase antienzyme 1, and tubulin.

### Statistical Analysis

We analyzed the behavioral data with the statistical program SPSS and followed significant effects (p < 0.05) with SPSS post-hoc contrasts with the repeated measures ANOVA. For the training and shock phases, the dependent variables were the number of methamphetamine or saline infusions during 20 training days and 10 footshock days. We analyzed RT-PCR data by one-way ANOVA followed by Fischer’s protected least-significant difference test (PLSD) using StatView (version 4.02, SAS Institute, Cary, NC, USA). Correlations between microarray and RT-PCR data were assessed using linear regression. The null hypothesis was rejected at p < 0.05.

## Additional Information

**How to cite this article**: Cadet, J. L. *et al.* Increased expression of proenkephalin and prodynorphin mRNAs in the nucleus accumbens of compulsive methamphetamine taking rats. *Sci. Rep.* **6**, 37002; doi: 10.1038/srep37002 (2016).

**Publisher’s note:** Springer Nature remains neutral with regard to jurisdictional claims in published maps and institutional affiliations.

## Supplementary Material

Supplementary Information

## Figures and Tables

**Table 1:** Classification of genes with altered expression after methamphetamine self-administration and footshocks in the NAc.

GenBank/RNA Accession number	Gene Symbol	Gene Name	Fold change ratio SR/CT	Fold change ratio SS/CT	Fold change ratio SR/SS
Membrane					
NM_001305460	Mpeg1	macrophage expressed 1	1.14	2.19	−1.92
Metabolism					
NM_001080756	Car12	carbonic anhydrase 12	1.47	−1.32	1.94
NM_001013882	Dctd	dCMP deaminase; 2 isoforms	−1.76	−1.22	−1.44
NM_001191849	Ndst4	N-deacetylase/N-sulfotransferase 4	−1.30	1.29	−1.67
NM_145084	Retsat	retinol saturase (all trans retinol 13,14 reductase)	−1.14	−2.69	2.37
Nucleic Acid Binding					
NM_001100653	Sidt1	SID1 transmembrane family, member 1	−1.44	1.16	−1.67
Protein Kinase/Phosphatase					
NM_019319	Mapk4	mitogen-activated protein kinase 4	1.37	−1.23	1.69
NM_080394	Reln	reelin	1.81	1.06	1.72
Signal Transduction					
NM_012750	Gfra2	GDNF family receptor alpha 2	−1.05	1.60	−1.68
NM_001106582	Gpr165	G protein-coupled receptor 165	−1.69	1.16	−1.96
NM_021680	Nxph4	neurexophilin 4	1.96	1.24	1.58
NM_019374	Pdyn	prodynorphin	2.56	1.12	2.28
NM_017139	Penk	proenkephalin	1.31	−1.30	1.70
NM_001107807	Pmepa1	prostate transmembrane protein, androgen induced 1	1.71	−1.05	1.80
NM_133568	Rasd2	RASD family, member 2	1.30	−1.29	1.67
NM_001106124	Slc39a12	solute carrier family 39 member 12	1.13	2.75	−2.42
NM_001124768	Tac1	tachykinin, precursor 1; 5 isoforms	1.04	−1.69	1.75
Synaptic Plasticity					
NM_031984	Calb1	calbindin 1	1.78	−1.03	1.83
NM_053988	Calb2	calbindin 2	−1.33	1.31	−1.75
NM_024140	Nrgn	neurogranin	1.76	−1.02	1.80
NM_030869	Nrp2	neuropilin 2	−2.00	−1.17	−1.70
Transcription					
XM_008770929	Dach1	dachshund family transcription factor 1	2.05	−1.01	2.07
NM_203410	Ifi27	interferon, alpha-inducible protein 27	2.12	1.85	1.15
NM_031069	Nell1	neural EGFL like 1	−1.49	1.16	−1.74
XM_008763702	Nr4a3*	nuclear receptor subfamily 4, group A, member 3	1.32	−1.32	1.75
unclassified					
777675	Ighd2-5	Mm immunoglobulin heavy diversity 2–5	1.28	−2.31	2.96

A gene was classified as significantly changed if it showed increased or decreased expression in SRvsCT, SSvsCT or SRvsSS comparisons according to an arbitrary cut-off at fold change ± 1.7, p < 0.05. Functional annotation and classification analyses for significantly changed genes were performed using DAVID Annotation tool^16^ (http://david.abcc.ncifcrf.gov) and were supported by literature searches.
