# Supplementary material for: Increased expression of proenkephalin and prodynorphin mRNAs in the nucleus accumbens of compulsive methamphetamine taking rats
Source: Sci Rep. 2016 Nov 14;6:37002. doi: 10.1038/srep37002 (PMC5108042; doi:10.1038/srep37002)
Supplement: Supplementary Information [file srep37002-s1.pdf]

Increased expression of proenkephalin and prodynorphin mRNAs in the nucleus accumbens of compulsive methamphetamine taking rats

Jean Lud Cadet*, Irina N. Krasnova, Donna Walther, Christie Brannock, Bruce Ladenheim, Michael T. McCoy , Daniel Collector, Oscar V. Torres, Ndeah Terry, and Subramaniam Jayanthi

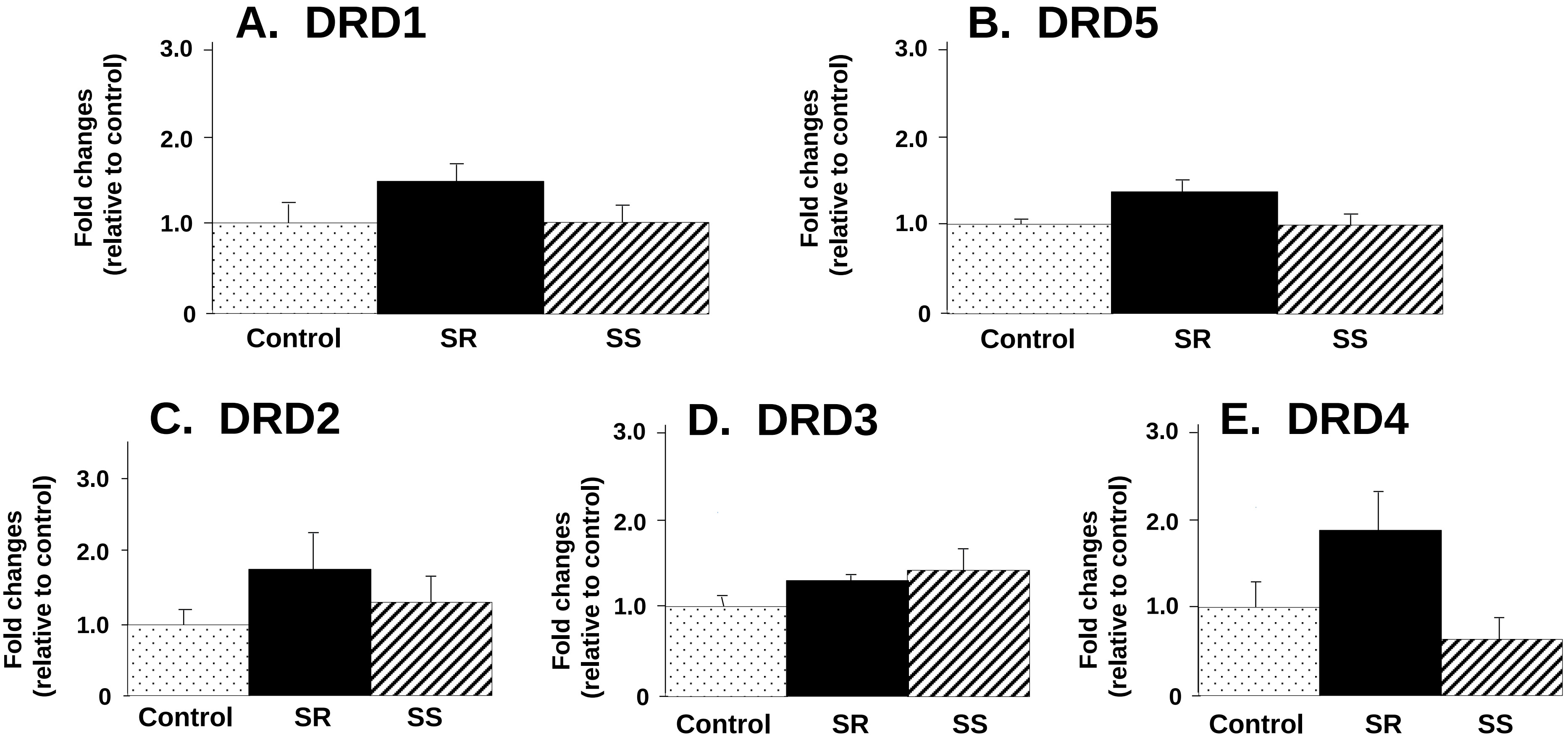

Supplementary Figure S1. Effects of methamphetamine self-administration and footshocks on the expression of dopamine receptors. qRT-PCR experiments were conducted as described in Fig. 4. Values are means + SEM in comparison to the control animals. (A-E) There were no significant changes in the expression of any of the five DA receptors between the groups. DRD1: $F(2, 15) = 1.658$, $p = 0.2236$; DRD2: $F(2, 14) = 1.192$, $p = 0.3326$; DRD3: $F(2, 15) = 1.255$, $p = 0.3133$; DRD4: $F(2,13) = 2.576$; $p = 0.1142$; DRD5: $F(2,13) = 1.739$, $p = 0.2094$.
